# Protocol for controlled gas-phase oxygen In-leakage in anaerobic bioreactors

**DOI:** 10.1016/j.mex.2026.104133

**Published:** 2026-08-31

**Authors:** Shota Shimokawara, Toshinari Maeda

**Affiliations:** a Electric Power Development Co., Ltd., 6-15-1 Ginza, Chuo-ku, Tokyo, 104-8165, Japan; b Department of Life Science and Systems Engineering, Kyushu Institute of Technology, Wakamatsu Campus, 2-4 Hibikino, Wakamatsu-ku, Kitakyushu, Fukuoka, 808-0196, Japan

**Keywords:** Gas chromatography, Mass flow controller, Oxygen contamination, Gas-fed fermentation

## Abstract

Trace oxygen may enter the gas supply line of an anaerobic bioreactor and affect reactor operation. In this study, we describe a method to expose a defined oxygen concentration for gas-fed anaerobic bioreactors. H_2_, CO_2_ and premixed CO_2_/O_2_ gas were supplied using mass flow controllers, and the reactor inlet O_2_ concentration was adjusted from 0 to 10,000 ppm. Both stepwise and constant exposure tests were carried out. The reactor outlet O_2_ concentration was measured by gas chromatography to confirm the actual exposure condition in bioreactors.

Because oxygen exposure is defined at the reactor inlet, stable exposure conditions can be applied regardless of oxygen consumption within the reactor.

This protocol provides an approach for investigating trace oxygen responses in continuously operated anaerobic microbial cultures.

• Defined inlet O2 concentrations can be generated by gas blending using mass flow controllers.
• Oxygen exposure conditions can be monitored by outlet gas chromatography.
• This method can be applied to stepwise and continuous oxygen exposure experiments.

## Graphical abstract

**Figure:**
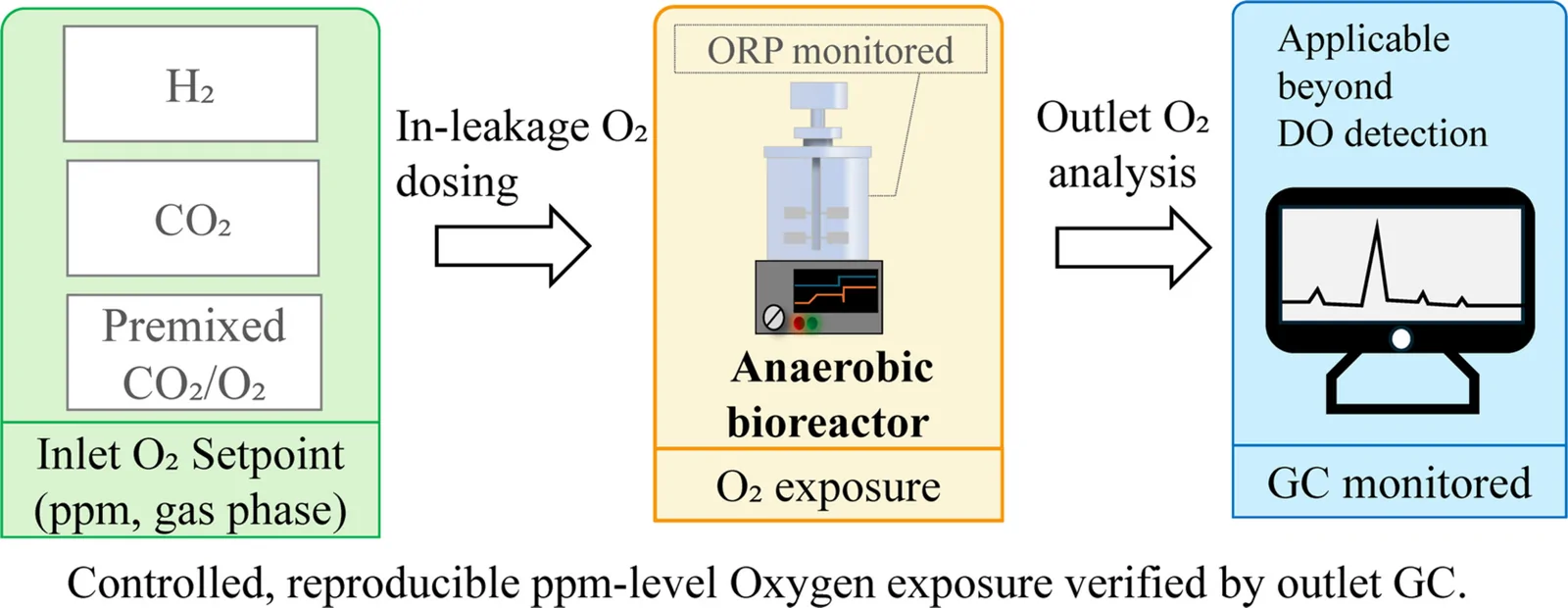

**Table:** 

**Subject area**	Environmental Science
**More specific subject area**	Anaerobic cultivation, Gas fermentation, oxygen exposure
**Name of your protocol**	Controlled Gas-Phase Oxygen In-leakage in Anaerobic Bioreactors
**Reagents/tools**	CO_2_ cylinder (≥99.999 % (v/v)) H_2_ cylinder (≥99.99 % (v/v)) CO_2_/O_2_ premixed cylinder (O_2_: 100–50,000 ppm(v/v) in CO_2_) Mass flow controllers (H_2_: 0–50 mL/min, CO_2_: 0–20 mL/min, premix: 0–20 mL/min, control range: 2–100 % of full scale, accuracy: ±1.0 % of full scale) Gas chromatograph (GC-990, GL Sciences, Japan) configured with two analytical channels: Channel 1: Molecular Sieve 5A column (10 m × 0.25 mm × 30 μm) with Ar as the carrier gas, used for H_2_, O_2_, CH_4_ analysis. Channel 2: PORAPLOT Q column (10 m × 0.25 mm × 8 μm) with He as the carrier gas, used for CO_2_ analysis. Thermal conductivity detector (TCD). ORP electrode (Redox Fermprobe S8, Broadley James, USA) 1000 mL Anaerobic bioreactor (BMZ02KP4, Biot, Japan) Soap film flowmeter (SF-1 U with Glass volume piping unit VP-1 U (0.2–10 mL/min), VP-2 U (2–100 mL/min), HORIBA, Japan) 6.35 mm SUS 316 L Tubing (OD: 6.35 mm, wall: 1.0 mm, ID: 4.35 mm) 3.18 mm SUS 316 L Tubing (OD: 3.18 mm, wall: 0.5 mm, ID: 2.18 mm)
**Experimental design**	*Methanothermobacter thermautotrophicus* DSM3590 was cultivated in a 1000 mL anaerobic jar fermenter at 60 °C, 600 rpm, and pH 7.0 according to the method of Martin et al. [1]. Oxygen exposure tests were conducted on a reactor that stabilized following 30 days of cultivation in pure H_2_ and CO_2_. Quantitative oxygen exposure tests were conducted by gradually introducing a known concentration CO_2_/O_2_ premixed gas into a CO_2_/H_2_ culture system using mass flow controllers. The response of microorganisms to oxygen exposure was measured as the methane production rate at the outlet of the reactor. Outlet gas composition and flow rate were quantified by gas chromatography and soap-film flowmeter. ORP was analyzed as a supplementary indicator of oxygen exposure.
**Trial registration**	Not applicable
**Ethics**	Not applicable
**Value of the Protocol**	● It is possible to test the effects of short-term and long-term oxygen exposure on microorganisms. ● Stable oxygen exposure conditions were provided for anaerobic bioreactors. ● Oxygen exposure can be evaluated without being affected by oxygen consumption in the bioreactor.

## Background

Batch culture experiments have been widely used to investigate oxygen exposure in anaerobic microorganisms [[2], [3], [4], [5]]. However, batch experiments are not sufficient for evaluating oxygen exposure events in continuously operated anaerobic bioreactors. Anaerobic bioreactors, particularly those operated over long periods, are likely to be exposed to continuous oxygen in-leakage.

In batch culture tests, oxygen in the gas-phase is rapidly consumed by microorganisms. Consequently, it is difficult to establish a test environment where the system is constantly exposed to a fixed amount of oxygen. It is possible to reduce the impact of oxygen consumption by periodically replacing the gas phase. However, because the oxygen consumption rate of methanogens is relatively high, this can be significantly burdened in practical experiments.

Although continuous oxygen exposure tests have recently been reported, reports are still limited [6]. Therefore, a method is needed that allows oxygen exposure to be defined under controlled conditions [7].

This protocol resolves these experimental challenges by continuously supplying a controlled amount of oxygen to anaerobic reactors. This could be a practical approach for evaluating microbial responses from controlled oxygen exposure. And it can be used for simulating oxygen in-leakage events in continuously operated anaerobic bioreactors.

## Description of protocol

### Purpose

This protocol is intended for continuously operating anaerobic gas-fed bioreactors, for example, bio methanation using H_2_ and CO_2_ as gaseous substrates. The protocol should be initiated only after stable condition of the bioreactor has been established. Oxygen exposure is achieved by introducing a premixed CO_2_/O_2_ gas into the H_2_/CO_2_ feed gas.

### Experimental setup

**Fig. 1:**
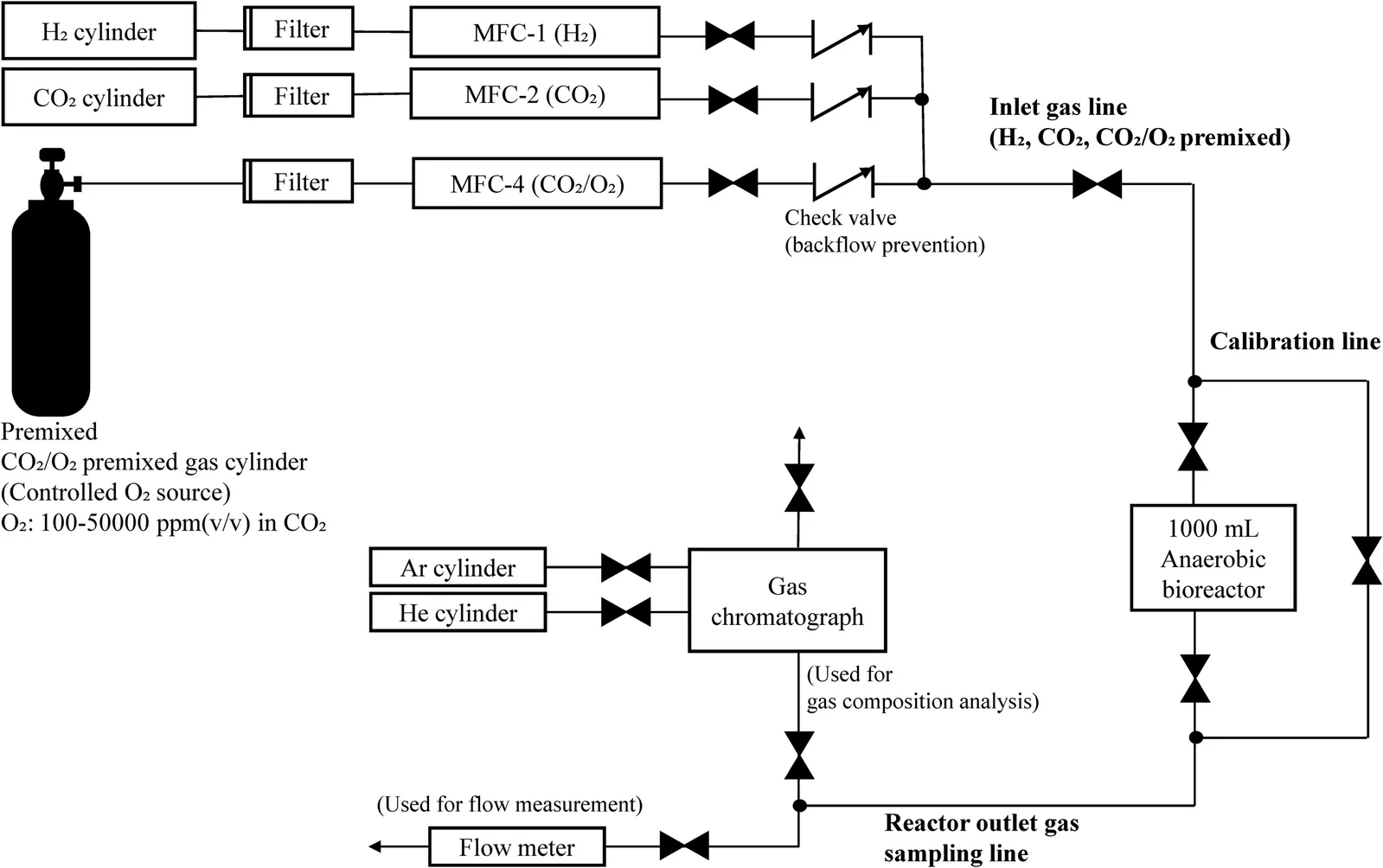
Schematic diagram of the experimental setup for controlled oxygen exposure in an anaerobic bioreactor.

A simplified schematic diagram of the experimental setup is shown in Fig. 1. Experimental setup consists of an anaerobic bioreactor, gas supply lines, and gas analysis equipment.

The bioreactor was operated under the conditions listed in Table 1. The reactor outlet gas was directed to the gas analyzer for measurement of CH_4_ and CO_2_ concentrations. Oxidation reduction potential (ORP) is recorded using an ORP electrode (Redox Fermprobe S8, Broadley James, USA). In this condition, bioreactor has been operated for 30 days until methane concentration and methane production rate at the reactor outlet reached stable values. Oxygen exposure experiments were initiated only after stable reactor performance had been confirmed.

**Table 1:** Bioreactor operating conditions.

Parameter	Value
Microorganism	*Methanothermobacter thermautotrophicus* DSM3590 (Leibniz Institute DSMZ, Braunschweig, Germany)
Reactor type	Stirred anaerobic bioreactor (BMZ02KP4, Biot, Japan)
Total Reactor volume	1000 mL
Working volume	700 mL
Cultivation Temperature	60 °C
pH	7.0
Agitation speed	600 rpm
H_2_ flow rate	40 mL/min
CO_2_ flow rate	10 mL/min

H_2_, CO_2_, and CO_2_/O_2_ premixed gas were supplied through mass flow controllers. The CO_2_/O_2_ premixed gas was used as an oxygen source, and inlet O_2_ concentrations were defined by adjusting the flow rates of CO_2_ and CO_2_/O_2_ gases. The gas supply lines were constructed from SUS316L tubing. The CO_2_/O_2_ premixed gas line was calibrated using a soap film flowmeter (SF-1 U, HORIBA, Japan) equipped with glass volume piping units (VP-1 U, HORIBA, Japan). Outlet gas composition was analyzed using gas chromatograph (GC-990, GL Sciences, Japan). O_2_, CH_4_, and CO_2_ concentrations were measured. The outlet gas was cooled to 10 °C and dried using a micro-GC dehumidifier (GL Sciences, Japan) before GC analysis.

The inlet O_2_ concentration was calculated from the corrected flow rate of the CO_2_/O_2_ premixed gas using the following equation:

$$C_{\text{in},O2}=\frac{C_{\text{premix}}F_{\text{premix}}}{F_{H2}+F_{\text{CO}2}+F_{\text{premix}}}$$

*C*_in, O2_ is the inlet O_2_ setpoint, *C*_premix_ is the O_2_ concentration in the premixed gas, and *F*_H2_, *F*_CO2_, and *F*_premix_ are the respective gas flow rates. The H_2_ flow rate was fixed at 40 mL/min. The combined flow rate of pure CO_2_ and the CO_2_/O_2_ premixed gas was maintained at 10mL/min.

### Experimental procedure

The inlet O_2_ setpoint was adjusted by replacing a portion of the pure CO_2_ gas with a CO_2_/O_2_ premixed gas. The total CO_2_ flow rate was kept constant, and the H_2_ flow rate was also kept constant under all experimental conditions. Therefore, the total gas flow rate remained constant throughout the experiments.

During the oxygen exposure experiments, outlet CH_4_ and O_2_ concentrations were measured at 15-min intervals by gas chromatography (GC). The ORP was continuously monitored throughout the experiments. The outlet gas flow rate was measured using a soap-film flow meter. The CH_4_ production rate was calculated from the outlet CH_4_ concentration and the outlet gas flow rate.

Immediately after changing the inlet O_2_ setpoint, a time lag occurred before the outlet gas composition reached the new setpoint because of the residual gas remaining in the reactor and gas lines. Therefore, the first GC measurement after each setpoint change was excluded from the data analysis.

CO_2_/O_2_ premixed gas containing 50,000 ppm O_2_ was used for the short-term oxygen exposure tests. At the start of the experiment, the CO_2_/O_2_ premixed gas was introduced while the pure CO_2_ flow rate was reduced by the same amount to supply the desired inlet O_2_ setpoint. The inlet O_2_ concentration increased stepwise from 0 to 10,000 ppm in eight setpoints. Each condition was maintained for 1 h. During each O_2_ exposure condition, the outlet CH_4_ and O_2_ concentrations were measured four times at 15-min intervals, and the outlet gas flow rate was measured once. After the 1 h exposure at the highest O_2_ concentration, the flow rate of CO_2_/O_2_ premixed gas was set to 0 mL/min, and the inlet O_2_ setpoint was returned to 0 ppm. The outlet CH_4_ and O_2_ concentrations were then monitored for 36 h to evaluate reactor recovery. For the long-term oxygen exposure tests, a CO_2_/O_2_ premixed gas containing 10,000 ppm O_2_ was used. The inlet O_2_ concentration was set to 600, 1000, 1200 ppm, and each condition was maintained for 48 h. During the exposure period, the outlet CH_4_ and O_2_ concentrations and the ORP were monitored, and the outlet gas flow rate was measured at three representative times for each condition. The concentrations were applied sequentially in the order of 600, 1000, 1200 ppm. Even when CH_4_ was no longer detected in the GC during oxygen exposure, the inlet O_2_ concentration was maintained until the end of the 48-h exposure test, and the ORP and outlet gas composition continued to be monitored.

## Protocol validation

**Fig. 2:**
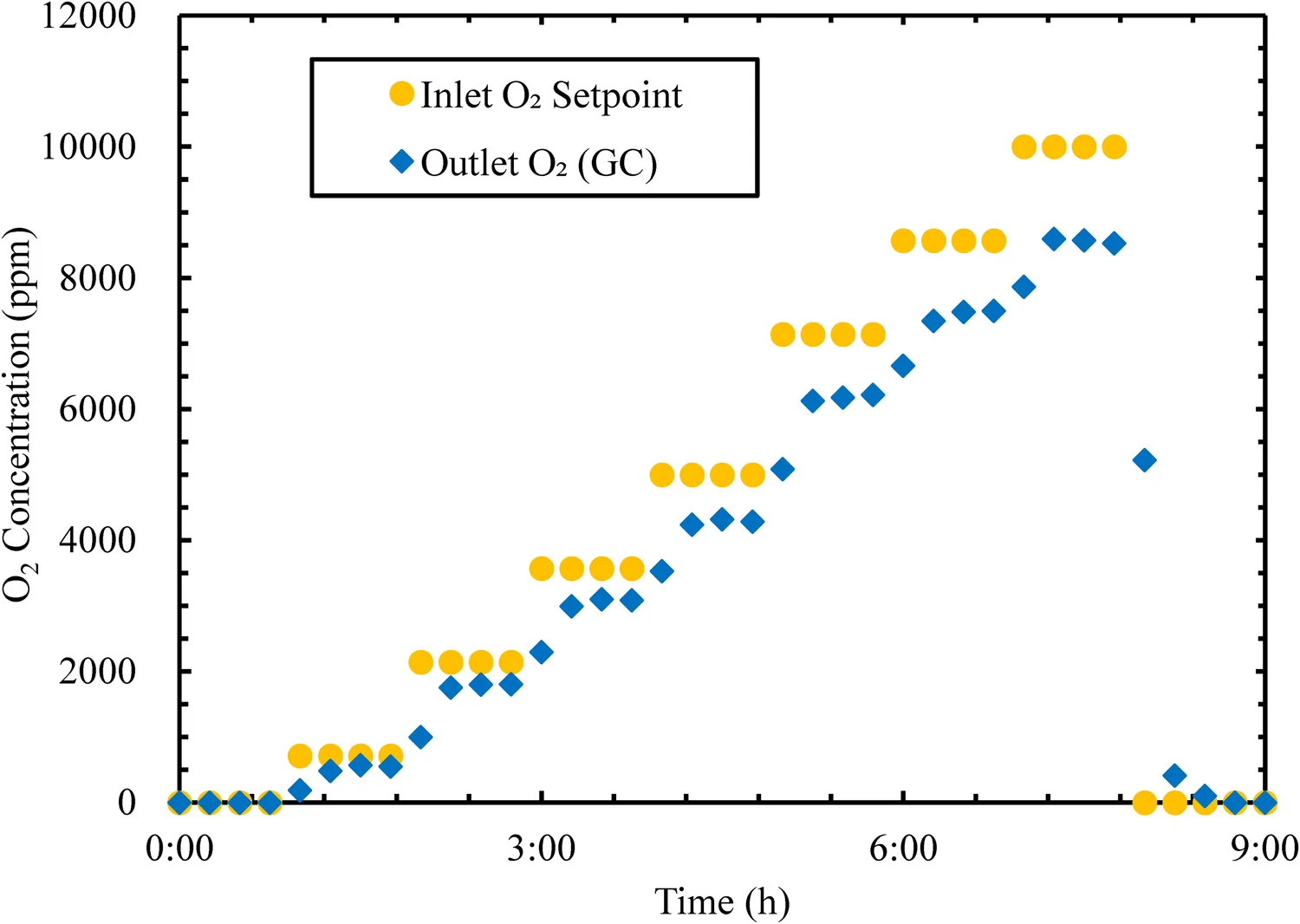
Representative result of the short-term oxygen exposure protocol. Showing the inlet O_2_ setpoints and outlet O_2_ concentrations.

Fig. 2 shows a representative result of the short-term oxygen exposure test using a 50,000 ppm O_2_/CO_2_ premixed gas. The inlet O_2_ setpoint was changed every 1 h, and the outlet O_2_ concentration followed each setpoint. Because oxygen was consumed within the reactor, the outlet O_2_ concentration remained lower than the inlet O_2_ setpoint. Except for the first measurement obtained 15 min after each setpoint change, the outlet O_2_ concentration reached a stable value for each inlet O_2_ setpoint. These results demonstrate that the protocol provided stable oxygen exposure conditions.

**Fig. 3:**
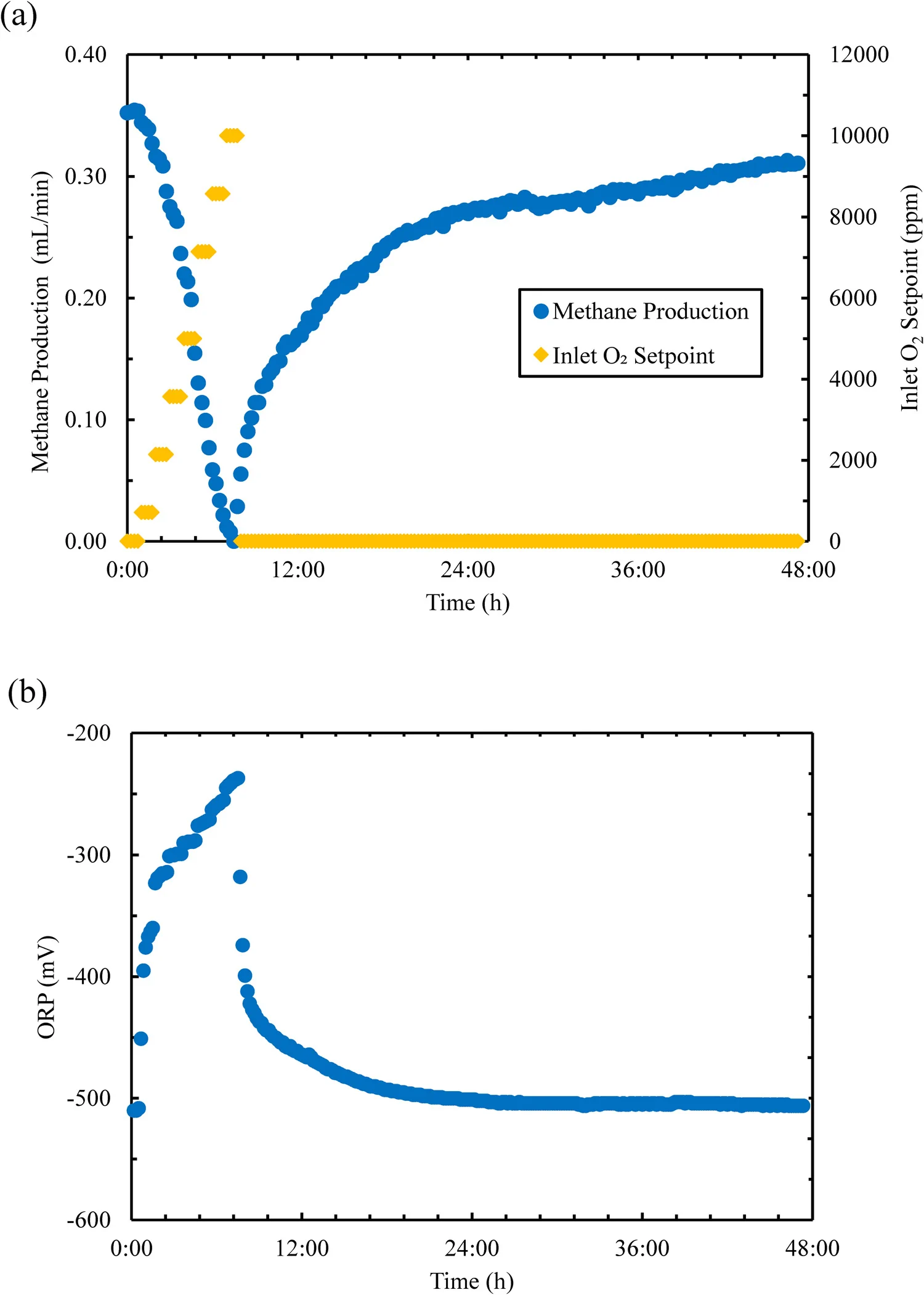
Result of the short-term oxygen exposure test. Methane production rate during stepwise oxygen exposure and reactor recovery. Oxidation-reduction potential (ORP) during stepwise oxygen exposure and reactor recovery.

Fig. 3(a) and 3(b) show results of the short-term oxygen exposure test. As the inlet O_2_ concentration was increased stepwise from 0 to 10,000 ppm, methane production decreased to 0 mL/min, while the ORP increased from −510 mV to −237 mV. After the inlet O_2_ setpoint was returned to 0 ppm following exposure to 10,000 ppm O_2_, methane production and ORP gradually recovered during the subsequent 36-h monitoring.

**Fig. 4:**
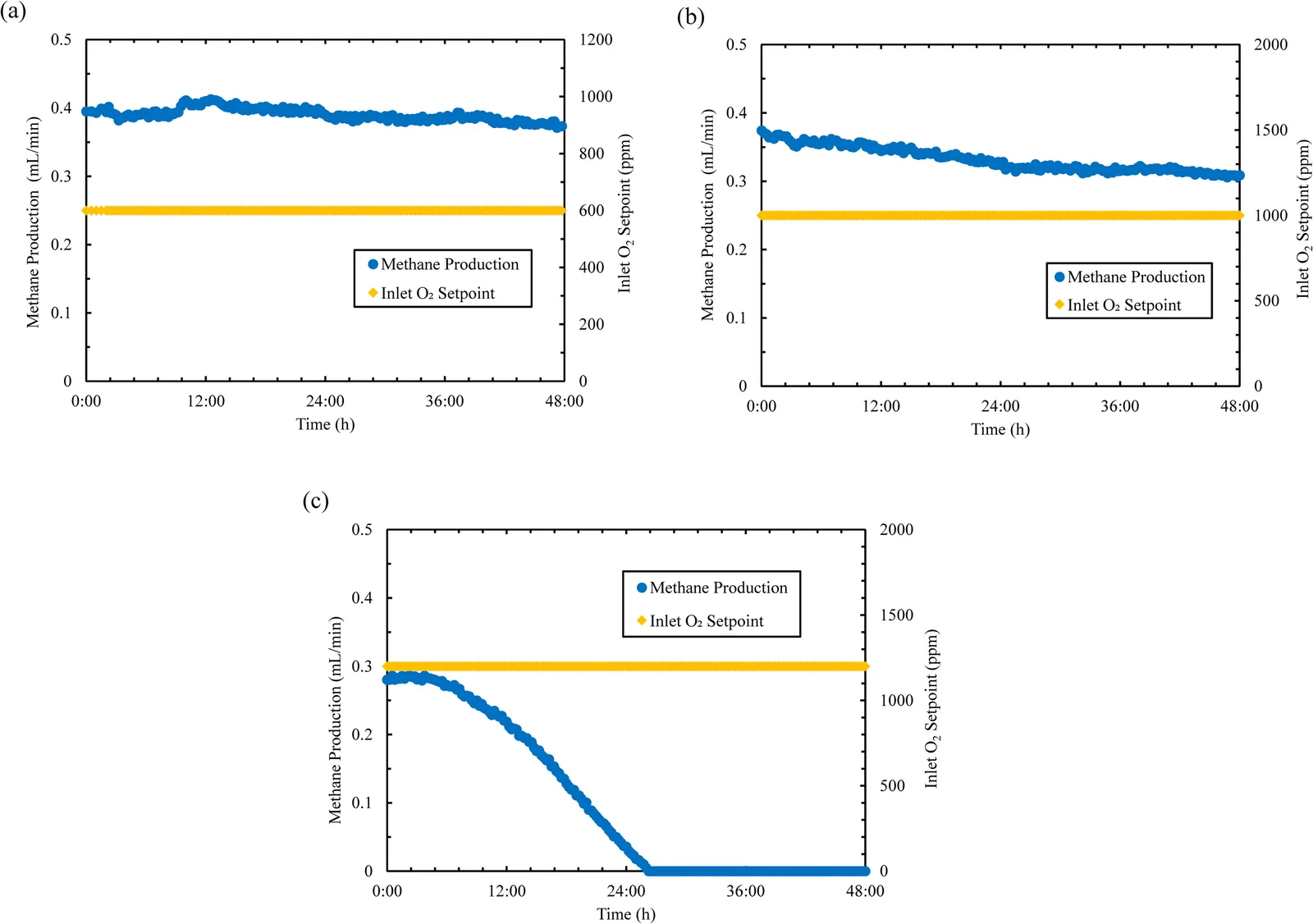
Results of the long-term oxygen exposure tests. Methane production rate during continuous exposure to 600 ppm inlet O_2_. Methane production rate during continuous exposure to 1000 ppm inlet O_2_. Methane production rate during continuous exposure to 1200 ppm inlet O_2_.

Fig. 4(a), 4(b), and 4(c) show results of the long-term oxygen exposure test at inlet O_2_ concentrations of 600, 1000, 1200 ppm. At an inlet O_2_ concentration of 1000 ppm, methane production gradually decreased during the 48-h exposure period. At 1200 ppm, methane production decreased to 0 mL/min after approximately 24 h and remained undetectable until the end of the test period. These results demonstrate that the long-term oxygen exposure protocol allows reactor performance to be monitored under continuous oxygen exposure for up to 48 h.

## Limitations

In this study, the short-term oxygen exposure test was performed at oxygen concentration of up to 10,000 ppm. The long-term oxygen exposure test was conducted at concentrations of up to 1200 ppm. Because the oxygen concentration that affects methanogenic activity is expected to vary depending on the microbial species and reactor operating conditions, preliminary oxygen exposure tests should be performed for each experimental system to determine an appropriate oxygen concentration range for evaluation.

## CRediT author statement

Shota Shimokawara: Conceptualization, Methodology, Investigation, Validation, Formal analysis, Data curation, Visualization, Writing – original draft, Writing – review & editing, Project administration.

Toshinari Maeda: Supervision, Writing – review & editing.

## Supplementary material *and/or* additional information [OPTIONAL]

None.

## Related research article

None.

## Declaration of competing interest

The authors declare that they have no known competing financial interests or personal relationships that could have appeared to influence the work reported in this paper.

## Data Availability

Data will be made available on request.
